# Does paracetamol improve quality of life, discomfort, pain and neuropsychiatric symptoms in persons with advanced dementia living in long-term care facilities? A randomised double-blind placebo-controlled crossover (Q-PID) trial

**DOI:** 10.1186/s12916-020-01858-6

**Published:** 2020-12-21

**Authors:** Paulien H. van Dam, Wilco P. Achterberg, Bettina S. Husebo, Monique A. A. Caljouw

**Affiliations:** 1 Department of Public Health and Primary Care, Leiden University Medical Centre, P.O. Box 9600, 2300 RC Leiden, the Netherlands; 2 Department of Global Public Health and Primary Care, Centre for Elderly and Nursing Home Medicine, University of Bergen, Bergen, Norway; 3 Municipality of Bergen, Norway

**Keywords:** Quality of life, Dementia, Paracetamol, QUALIDEM, Long-term care facility

## Abstract

**Background:**

The objectives of this study are to determine the effects of regularly scheduled administration of paracetamol (acetaminophen) on quality of life (QoL), discomfort, pain and neuropsychiatric symptoms of persons with dementia living in long-term care facilities (LTCFs).

**Methods:**

A multicentre randomised double-blind placebo-controlled crossover trial for 13 weeks (January 2018 to June 2019) in 17 LTCFs across the west of the Netherlands. Inclusion criteria were age ≥ 65 years, (advanced) dementia and a moderate to low QoL, independent of the presence of pain (QUALIDEM ≤ 70). Exclusion criteria were the use of regular pain treatment, allergies to the study medication, severe liver disease, use of > 4 units of alcohol/day, weight < 50 kg and/or concomitant use of flucloxacillin. Participants received study medication (paracetamol/placebo) in two periods of 6 weeks each (1 week in between as a wash-out period). Randomisation decided in which order participants received paracetamol and placebo. Primary outcomes included QoL (QUALIDEM) and discomfort (DS-DAT), secondary outcomes included pain (MOBID-2) and neuropsychiatric symptoms (NPI-NH).

**Results:**

Ninety-five LTCF residents (mean age 83.9 years [SD 7.6], 57.9% females) were included. Repeated linear mixed models showed no difference in mean differences of QUALIDEM (paracetamol +1.3 [95% CI -1.0–3.5], placebo +1.5 [95% CI -0.7–3.8]), DS-DAT (paracetamol -0.1 [95% CI -1.4–1.2], placebo 0.6 [95 CI -0.7–1.8]), MOBID-2 (paracetamol 0.0 [95% CI -0.5–0.5], placebo -0.2 [95% CI -0.7–0.3]) and NPI-NH (paracetamol +1.5 [95% CI -2.3–5.4], placebo -2.1 [95% CI -6.0–1.7]) in favour of either paracetamol or placebo.

**Conclusions:**

Compared to placebo, paracetamol showed no positive effect on QoL, discomfort, pain and neuropsychiatric symptoms in persons with advanced dementia with low QoL. It is important to find out more specifically which individual persons with advanced dementia could benefit from pain treatment with paracetamol, and for clinicians to acknowledge that a good assessment, monitoring and multidomain approach is vital for improving QoL in this vulnerable group.

**Trial registration:**

Netherlands Trial Register NTR6766. Trial registration date: 20 October 2017

## Background

The expected increase in the number of persons with dementia in future decades [1] emphasises that caregivers need to be able to cope with the difficulties they experience daily in order to maintain optimal quality of life (QoL) in this population. The focus on QoL has become more and more pronounced in recent decades, but as the persons with dementia are mostly unable to adequately indicate how they experience their QoL, the intricate task of safeguarding it for them falls to the people around them [2]. In a long-term care facility (LTCF), there are even more (professional) caregivers who are responsible for the maintenance and/or improvement of the QoL of these persons.

Two of the principal goals proposed by the World Health Organization in their recent factsheet on dementia to improve the lives of persons with dementia are to optimise well-being and to identify and treat physical and psychological problems [1]. The latter category contains many factors that may be negatively associated with the QoL of a person with dementia, including the presence of depression, behavioural problems, pain, comorbidity, living alone and having needs that are unmet [3, 4]. The strength and direction of these associations, however, vary considerably between individuals [5]. One of the mentioned factors, pain, can be treated. However, there is still a group of persons with dementia that have undiagnosed and therefore untreated pain. Untreated, it may be associated with neuropsychological problems, e.g. behavioural problems (agitation, aggression, psychosis) [6–9] and depression [9, 10]. On the other hand, in view of the large increase in opioids and paracetamol prescription in the past years [11–14], clinicians should be aware of side effects and overtreatment with pain medication in this population.

The use of pain medication has been proven effective on agitation [15, 16], depression and apathy [17], sleep [18] and social interaction [19] in persons with dementia. Two relatively small trials with a crossover design were performed earlier to assess the effects of pain medication (paracetamol) in this target population. One included 25 participants (mean age 85.9 years, 88% female) living in LTCFs in which the authors concluded that paracetamol improved social interaction [19]. The second study included 39 participants (mean age 85.7, 87% female, mean Global Deterioration Score 5.7) living in LTCFs [20]. The researchers of this study found no significant difference in discomfort between the placebo and paracetamol groups. However, so far, no studies have investigated the effect of paracetamol on overall QoL of persons with dementia. The question remains whether paracetamol only has analgesic and antipyretic effects [21], or also other (unknown) effects that may influence QoL in persons with advanced dementia. Therefore, the present study aims to investigate the effect of regularly scheduled administration of paracetamol (acetaminophen) on QoL of persons with dementia with low QoL, independent of having pain, living in LTCFs. Furthermore, the effect of scheduled administration of paracetamol on discomfort, pain and neuropsychiatric symptoms will be assessed [22].

## Methods

From January 2018 to June 2019, we performed a multicentre (block) randomised double-blind placebo-controlled crossover trial for 13 weeks in LTCFs connected to the University Network of the Care sector South Holland (UNC-ZH) in the west of the Netherlands [22]. The UNC-ZH is a collaboration between the Leiden University Medical Center (LUMC) and large care organisations in the west of the Netherlands. Its goal is to initiate, facilitate and perform care-related scientific research [23].

### Participants and enrolment

This study aimed to include 95 LTCF residents aged ≥ 65 years, with (advanced) dementia stage 5, 6, or 7 according to the Reisberg Global Deterioration Scale [24] and a moderate to low QoL, total score ≤ 70 on QUALIDEM-6-Domain total score (QUALIDEM-6D), independent of having pain. This cut-off point was derived from the median of the QUALIDEM-6D scores found in a previous Dutch study involving persons with dementia living in LTCFs [25, 26].

Exclusion criteria were use of regular pain treatment (residents who used paracetamol that was prescribed 'pro re nata', or 'as needed' (PRN) were eligible only if the use of paracetamol in the week previous to starting study medication was ≤ 3 g/week with a maximum of 1 g/day), allergies to the study medication (paracetamol or placebo), severe liver insufficiency or disease, use of > 4 units of alcohol/day, weight < 50 kg and/or concomitant use of flucloxacillin [27].

### Intervention

Study medication was produced and provided by the pharmacy of the LUMC. Participants received study medication in two periods of 6 weeks each with 1 week in between as a wash-out period. One period consisted of paracetamol, the other of placebo. In accordance with a Dutch guideline for chronic use of paracetamol in older persons, the dose of paracetamol in the first 4 weeks was slightly higher (3 times/day 1000 mg) than the last 2 weeks of this period (2 times/day 1000 mg and 1 time/day 500 mg) [28]. Placebo tablets were provided in the same amount and resembled the paracetamol tablets in appearance, taste and composition. The bitter taste was imitated by adding a low dose of quinine (without therapeutic activity) to the placebo substance. The study medication was packaged in identical jars and administered to the participants along with their other medication by nurses and nursing assistants that were allowed to administer medication, in the same way they were used taking their medication. When, however, pain treatment was needed, a single administration of paracetamol 1000 mg was allowed without consequences, but no more than 3 times/week. When more pain treatment was needed, the participant stopped study medication, but the measurements continued, following the intention-to-treat principle.

### Randomisation, treatment allocation and blinding

Included participants were randomised in blocks of 4 by a random number generator in the pharmacy of the LUMC. Participants were randomised 1:1 into the paracetamol-placebo (AB) or the placebo-paracetamol (BA) treatment arm. Participants and their informal caregivers, researchers, research nurse and professional caregivers in the participating LTCFs were blinded to treatment allocation. Only the pharmacy of the LUMC knew which participant was allocated to which treatment arm.

### Outcome measures

All data concerning the primary and secondary outcomes listed below were collected at baseline, 6 weeks, 7 weeks and 13 weeks. QoL, discomfort and pain were observed by the responsible nurse or nursing assistants, and neuropsychological symptoms were measured via interviews with the nurse/nursing assistant by a research nurse.

### Primary outcomes

#### Quality of life and discomfort

The short 18-item version of the QUALIDEM was used to measure QoL. This version comprises six domains (care relationship, positive affect, negative affect, restless tense behaviour, social relationships and social isolation) that are also applicable to persons with very severe dementia [29, 30]. In order to calculate a total mean score for QoL, the individual domain scores were re-calculated to a percentage score by dividing the domain score by its maximum achievable points multiplied by 100. Domain scores were then added up and divided by 6 to calculate an overall mean score, the QUALIDEM-6D. Both the domain scores and the overall mean score can range from 0 (worst QoL possible) to 100 (best QoL possible). These transformations have been applied successfully multiple times in previous studies [12, 31–33].

The Discomfort Scale-Dementia of Alzheimer Type (DS-DAT) was used to measure discomfort in persons with advanced dementia [34]. It consists of nine items of discomfort with a score ranging from 0 (no discomfort) to 27 (worst possible discomfort).

### Secondary outcomes

#### Pain

The nurse/nursing assistant observed pain in the participants during morning care using the Mobilization-Observation-Behaviour-Intensity-Dementia-2 pain scale (MOBID-2) [35, 36]. This observational instrument has been proven reliable, valid and very responsive to change of pain in persons with dementia [35, 37]. While moving hands, arms and legs of the participant, turning the participant on both body sides on the bed and letting him/her sit on the edge of the bed, the nurse/nursing assistant rated pain intensity by observing facial expressions, vocalisations and defending behaviour. Subsequently, the nurse/nursing assistant rated pain intensity by observation based on pain behaviour over the preceding week related to head/neck, chest/lungs/heart, upper abdomen, legs/pelvis/lower abdomen and skin/wounds. A total pain score ranging from 0 (no pain) to 10 (worst pain possible) was assigned to these observations. Scores ≥ 3 were seen as clinically relevant pain [37].

#### Neuropsychiatric symptoms

Neuropsychiatric symptoms were measured with the Neuropsychiatric Inventory—Nursing Home version (NPI-NH) [38, 39]. This is an interview-based questionnaire completed by the nurse/nursing assistant and the research nurse, consisting of 13 items that are each scored for frequency and severity. Total scores range from 0 (no behavioural problems) to 144 (very severe behavioural problems).

### Additional measurements at baseline

Demographic data were collected at the start of the study by the nursing staff and the treating elderly care physicians. The severity of dementia was measured with the Reisberg Global Deterioration Scale (GDS), which reflects the stage of progression of the disease from 1 (no cognitive decline) to 7 (very severe cognitive decline) [24]. Comorbidity was assessed using the Functional Comorbidity Index, a list of 18 comorbid diseases that are associated with physical function [40].

### Compliance

The participants’ compliance to study medication was tracked by counting residual study medication after each finished study period. A leftover tablet count of > 10% (> 24 missed tablets) per period was considered non-compliant. Also, the medication intake was registered on a medication registration form by the nurse/nursing assistant each time the (study) medication was administered. When participants refused study medication repeatedly, the nurse/nursing assistant informed the researchers and the study medication was discontinued on the medication administration form. The same applied to participants who had to stop because of starting (other) pain medication.

### Sample size calculation

A sample size of 70 participants was calculated based on the detection of an inter-individual difference of 10% on the primary outcome measure QUALIDEM, with 80% power, and alpha 0.05. To account for an estimated dropout of 35% (mortality, loss to follow-up, (other) pain medication needed, etc.), enrolment of 95 participants was planned.

### Statistical analysis

At baseline, the characteristics and outcome measures in the two different treatment arms were compared using unpaired *t* tests for normally distributed numerical data, one-way ANOVA tests for non-normally distributed data and chi-squared tests for categorical data.

The decision which statistical tests to use was based on whether the main outcome measure QUALIDEM showed an order and/or period effect. The calculation of these effects was extensively described in the protocol article of this study [22]. If no significant order and/or period effect was found, the two treatment groups, i.e. placebo and paracetamol, would be compared using paired *t* tests for normally distributed numerical data, Wilcoxon signed-rank tests for non-normally distributed data and chi-squared tests for categorical data. In case of any order and/or period effect, repeated linear mixed models were used with adjustment for order and/or period effects.

### Patient and public involvement

The topic of our study was identified by the quality of life feedback group, in which care professionals of LTCFs participate. The members of the UNC-ZH (care organisations), combined with the client panel of older people from the LUMC and the QoL feedback group, felt that they needed feasible and evidence-based interventions that could help achieve optimal QoL in persons with (advanced) dementia. Therefore, they provided input to the researchers and the UNC-ZH to develop this study. The study was subsequently designed and performed in co-creation with these three groups.

## Results

### Enrolment and study flow

**Fig. 1:**
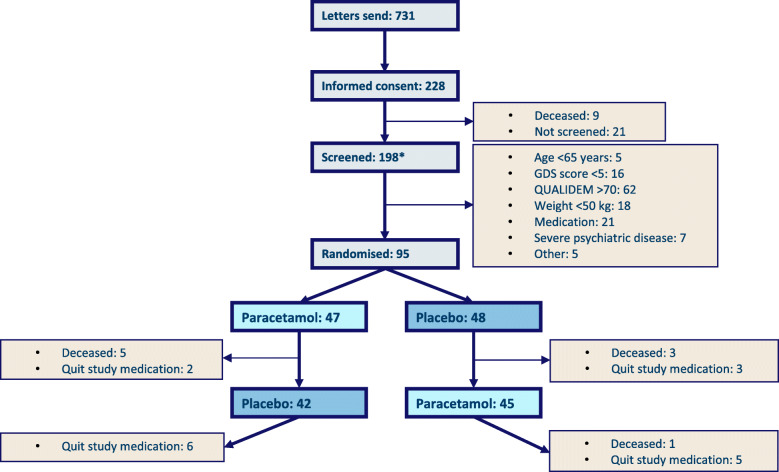
Flowchart of the Q-PID trial * Some overlap exists in the number of stated reasons for exclusion, because some persons met more than 1 exclusion criterium

A total of 731 patient information letters were sent to legal representatives of eligible participants. Of these legal representatives, 228 consented to screening. Nine persons in this screening group died before enrolment/randomisation, and 21 persons were not screened because the planned number of 95 participants was reached. One hundred ninety-eight eligible participants were eventually screened for inclusion and exclusion criteria. The main reasons for exclusion were a QUALIDEM > 70 (62 persons), using pain medication and/or medication interacting with study medication (21 persons), weight < 50 kg (18 persons) and a GDS score below 5 (16 persons). All reasons for exclusion can be found in Fig. 1. Finally, 95 LTCF residents with advanced dementia across 17 LTCFs (9 care organisations) in the west of the Netherlands were enrolled in this study: 47 in the paracetamol-placebo (AB) arm and 48 in the placebo-paracetamol (BA) arm.

During the study, 9 participants died (not study-related), of whom 8 in the first study period.

### Baseline characteristics of participants

The mean age of the participants was 83.9 years (SD 7.6), 57.9% were female, the majority had a GDS score of 6 (70.5%) and the mean number of comorbidities according to the FCI in the total group was 2.7 (SD 2.0). These participant characteristics did not differ at baseline across both treatment arms (Table 1).

**Table 1:** Baseline characteristics and measurements of the total group, stratified by randomisation group

	Paracetamol-placebo, ***N*** = 47	Placebo-paracetamol, ***N*** = 48
**Mean age (SD) in years**	83.9 (7.5)	83.9 (7.7)
**Female (%)**	27 (57.4)	28 (58.3)
**GDS score 7 (%)**	10 (21.3)	10 (20.8)
**FCI, 0–18 (SD)**	2.9 (1.9)	2.5 (2.1)
**QUALIDEM-6D**		
*Total score 0–100 (SD)*	58.1 (13.1)	57.0 (13.8)
*A—Care relationship 0–100 (SD)*	58.0 (22.3)	56.9 (23.0)
*B—Positive affect 0–100 (SD)*	69.6 (18.6)	68.4 (19.7)
*C—Negative affect 0–100 (SD)*	63.8 (28.0)	64.2 (25.0)
*D—Restless tense behaviour 0–100 (SD)*	37.9 (25.5)	39.8 (28.6)
*F—Social relationships 0–100 (SD)*	64.0 (21.2)	58.8 (20.9)
*G—Social isolation 0–100 (SD)*	55.1 (20.8)	53.7 (23.7)
**DS-DAT, 0–27 (SD)**	8.4 (4.9)	8.3 (6.0)
**Pain (MOBID-2 ≥ 3) (%)**	15 (33.3)*	15 (31.3)
**MOBID-2 overall pain intensity, 0–10 (SD)**	2.0 (2.4)	2.3 (3.0)
**NPI-NH**		
*Total score, 0–144 (SD)*	32.6 (21.0)	33.5 (18.9)
*Psychosis 0–24 (SD)*	3.7 (5.8)	3.7 (4.6)
*Agitation 0–48 (SD)*	10.7 (8.6)	11.9 (9.7)
*Affective symptoms 0–24 (SD)*	5.9 (6.2)	4.8 (5.7)
**No psychotropic use**^**†**^ **(%)**	29 (61.7)**	19 (39.6)**

*SD* standard deviation, *GDS* Global Deterioration Scale, *FCI* Functional Comorbidity Index, *QUALIDEM-6D* dementia-specific QoL measurement instrument, 6 domain version, *DS-DAT* Discomfort Scale-Dementia of Alzheimer Type, *MOBID-2* Mobilization-Observation-Behaviour-Intensity-Dementia-2 pain scale, *NPI-NH* Neuropsychiatric Inventory—Nursing Home version

*Missing, 2

**p value 0.031 (Pearson chi-square)

^†^Psychotropics: antipsychotics, antidepressants, anxiolytics, hypnotics and anti-dementia drugs

### Order and period effects

Comparing the treatment effects of paracetamol on the QUALIDEM of both groups in both periods, i.e. the effect of paracetamol minus the effect of placebo, revealed a significant difference in mean differences of the QUALIDEM total scores between the two treatment arms (4.5 in the AB arm and -4.8 in the BA arm; *p* = 0.008), which means that there was an order effect in the main outcome measure QUALIDEM.

### Primary outcomes

#### Quality of life

**Fig. 2:**
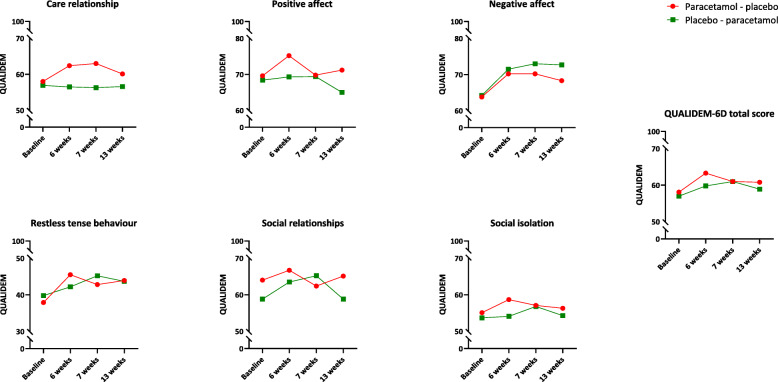
Mean QUALIDEM domain scores and mean QUALIDEM-6D total scores in the two treatment groups during the Q-PID study *QUALIDEM*, questionnaire to measure QoL in persons with dementia, range 0 (worst QoL) to 100 (best QoL); *QUALIDEM-6D*, 6-domain total score of the QUALIDEM questionnaire, range 0 (worst overall QoL) to 100 (best overall QoL); *paracetamol-placebo*, baseline to 6 weeks paracetamol, 7 to 13 weeks placebo; *placebo-paracetamol*, baseline to 6 weeks placebo, 7 to 13 weeks paracetamol

At baseline, the groups in the two treatment arms did not differ on QUALIDEM-6D total scores (AB arm 58.1 [SD 13.1] vs. BA arm 57.0 [SD 13.8]; *p* = 0.701) and the six QUALIDEM domain scores (Table 1). The QUALIDEM-6D scores of each treatment arm during the study are shown in Fig. 2.

A strong period effect, i.e. the mean changes in both periods in the total group of participants were significantly different, was found for the QUALIDEM-6D total score (+3.8 in period 1 vs. -1.0 in period 2; *p* = 0.004), and the subdomain negative affect (6.7 in period 1 vs. -1.2 in period 2; *p* = 0.005).

Application of repeated linear mixed models subsequently showed no differences in the QUALIDEM-6D total scores and domain scores in favour of either paracetamol or placebo (Table 2).

**Table 2:** Treatment effects of paracetamol and placebo on quality of life, discomfort, pain and neuropsychiatric symptoms. *N* = 95 (baseline), *N* = 86 (end of study)

	Intervention	Mean difference	95% CI	***p*** value
**QUALIDEM-6D**^**†**^				
*Total score*	Paracetamol	1.3	-1.0–3.5	0.876
	Placebo	1.5	-0.7–3.8	
*A—Care relationship*	Paracetamol	2.9	-1.0–6.9	0.128
	Placebo	-1.4	-5.3–2.5	
*B—Positive affect*	Paracetamol	0.3	-3.7–4.3	0.872
	Placebo	0.7	-3.2–4.7	
*C—Negative affect*	Paracetamol	2.6	-1.4–6.6	0.919
	Placebo	2.9	-1.0–6.9	
*D—Restless tense behaviour*	Paracetamol	2.1	-3.1–7.3	0.955
	Placebo	2.3	-2.8–7.5	
*F—Social relationships*	Paracetamol	-1.1	-5.3–3.1	0.192
	Placebo	2.9	-1.3–7.0	
*G—Social isolation*	Paracetamol	0.9	-3.6–5.3	0.803
	Placebo	0.1	-4.3–4.5	
**DS-DAT**^‡^	Paracetamol	-0.1	-1.4–1.2	0.478
	Placebo	0.6	-0.7–1.8	
MOBID-2*	Paracetamol	0.0	-0.5–0.5	0.605
	Placebo	-0.2	-0.7–0.3	
**NPI-NH**^**††**^				
*Total score*	Paracetamol	1.5	-2.3–5.4	0.187
	Placebo	-2.1	-6.0–1.7	
*Psychosis*	Paracetamol	-0.3	-1.4–0.8	0.935
	Placebo	-0.3	-1.4–0.8	
*Agitation*	Paracetamol	1.2	-0.7–3.0	0.077
	Placebo	-1.2	-3.0–0.7	
*Affective symptoms*	Paracetamol	-0.3	-1.5–0.9	0.516
	Placebo	0.2	-0.9–1.4	

Repeated linear mixed models, adjusted for order and period effects, and psychotropic use

*CI* confidence interval, *QUALIDEM-6D* dementia-specific QoL measurement instrument, 6 domain version, *DS-DAT* Discomfort Scale-Dementia of Alzheimer Type, *MOBID-2* Mobilization-Observation-Behaviour-Intensity-Dementia-2 pain scale, *NPI-NH* Neuropsychiatric Inventory-Nursing Home version

*Higher score means more pain

^†^Higher score means better QoL

^‡^Higher score means more discomfort

^††^Higher score means more neuropsychiatric symptoms

#### Discomfort

The groups in the two treatment arms did not differ on DS-DAT total scores at baseline (AB arm 8.4 [SD 4.9] vs. BA arm 8.3 [SD 6.0]; *p* = 0.970). No difference was found in the treatment effects of paracetamol and placebo (paracetamol -0.04 [95% CI -1.3–1.3] vs. placebo 0.6 [95% CI -0.7–1.9]) (Table 2).

### Secondary outcomes

#### Pain

Mean MOBID-2 pain scores at baseline were similar in both treatment arms (AB arm 2.0 [SD 2.4] vs. BA arm 2.3 [SD 3.0]; *p* = 0.531) (Table 1). No difference in treatment effect on pain was found between both treatments (paracetamol 0.0 [95% CI -0.5–0.5], placebo -0.2 [95% CI -0.7–0.3]) (Table 2).

#### Neuropsychiatric symptoms

At the start of the study, there was no significant difference in NPI-NH total mean scores between the groups in the two treatment arms (AB arm 32.6 [SD 21.0] and BA arm 33.5 [SD 18.9]; *p* = 0.822) and the three subdomain scores (psychosis 3.7 [SD 5.8] vs. 3.7 [SD 4.6], *p* = 0.974; agitation 10.6 [SD 8.6] vs. 11.9 [SD 10.0], *p* = 0.512; affective symptoms 5.9 [SD 6.2] vs. 4.8 [SD 5.7], *p* = 0.396) (Table 1). No difference in treatment effect between paracetamol and placebo was found for the NPI-NH total mean score and the three subdomain scores (Table 2).

### Compliance

Five participants quit study medication in the first period and 11 in the second study period. Reasons for quitting study medication were repeated refusal of study tablets (7 participants) and being in need of (other) pain medication (9 participants). Two participants from the latter group performed much better in the first period, and the nurses detected a clear deterioration in the second period. This caused them to contact the researchers to quit study medication and to continue paracetamol (although unsure which treatment arm the participant was in, the difference between the two periods was evident). After the study ended and after deblinding, these participants indeed turned out to be part of the AB treatment arm (first paracetamol, then placebo). At least two other patients did not stop study medication, but the nurses again saw a clear difference and when paracetamol was continued after the study ended, the participants performed better and were more relaxed.

In the first study period, the median compliance was 92.0% (IQR 80.7–100.0), taking into account participants who died and who stopped study medication during this period. Data for 14 participants on the number of residual tablets at the end of the first period was missing, due to the absence of the study medication jars on the LTCF units after the study period ended. In the second study period, the median compliance was 84.0% (IQR 67.5–98.1), taking into account participants who died and who stopped study medication during this and previous study period. Compliance data for 18 participants was missing for the same reason as in period 1. The medication administration records showed better compliance than the residual tablet counting, indicating an imbalance between recording the study medication as ‘given’ and actually giving it.

## Discussion

The present study shows that paracetamol, compared to placebo, did not improve QoL, discomfort, pain and neuropsychiatric symptoms in persons with advanced dementia. It is important to take a closer look at the appropriateness of prescribing pain medication in these vulnerable persons. Also, doctors need to be aware that medication for sleep and neuropsychiatric symptoms has side effects and that (undertreated) pain may be the cause of sleep problems and/or neuropsychiatric symptoms, as has been described by others [41].

Several strengths and limitations can be mentioned. First, to our knowledge, this is the largest crossover study with persons with dementia performed in LTCFs. The crossover design is an efficient study design that requires a substantially smaller sample size than trials with parallel groups [42, 43], causing less variance between measurements. Consequently, up to four times less participants are needed to reach the same power as a parallel group study. In view of the target group, this was an important consideration in choosing the design for this study. Moreover, the crossover design is very suitable when a wash-out period longer than five times the halftime of the intervention can be fitted into the study design, so that no carry-over effects are expected after stopping or changing intervention. Also, confounding is minimised since the participants are their own controls and baseline characteristics will therefore be equal.

Prior to the study, we were aware, due to previous experiences in research in this field, that only approximately 10% of all persons with dementia living in the participating LTCFs would be eligible to participate. Therefore, a lot of effort was needed and made in the present study to achieve the goal of enrolling 95 participants, which succeeded within the planned time frame. Furthermore, the study was performed within the care organisations that are member of the UNC-ZH, which assures a good research infrastructure.

Obviously, research in persons with advanced dementia living in LTCFs is complex and does not resemble research in a preconceived environment. One of the prerequisites to perform a crossover study is that the disease that will be studied is chronic and has a stable course. As we saw in our results, the entire group of participants performed worse in the second study period, irrespective of which treatment arm they were in. Therefore, we used mixed effects models accounting for this period effect, rather than simply comparing the treatment groups crosswise. It may be possible that the dementia (and other comorbidities) deteriorated during the course of the study, which may have caused worse outcomes in the second period. We did not record the course of progression of the dementia and comorbidities during follow-up. It is also possible that the workload accompanying the present study caused the nurses/nursing assistants to be less motivated in the second period, contributing to the period effects found for the QUALIDEM and the NPI-NH.

Second, the time frames in which questionnaires needed to be completed at each time point were tight, so it was not always feasible to have the same nurse/nursing assistant complete the questionnaires at all time points for the same participant. Although we used questionnaires that are thoroughly validated, there is always a component of subjectivity, in which the connection between the person with dementia and the nurse/nursing assistant is important for how the questionnaires are completed.

Third to mention is the compliance of the study medication. The number of participants that quit study medication as reported by nursing staff was within our a priori estimated ‘quit rate’ (35% of 95 participants), but when counting the remaining tablets after each period, more participants appeared to have not received their study medication according to our definition of compliance (missed < 10% of tablets). Nevertheless, the median compliance in both periods was still 92.0% and 84.0%. The study medication could not be provided in the same unit dose packages as the other medication, due to logistical problems between the separate preferred pharmacies in 17 nursing homes, and the additional costs associated with the organisation of this method/finances. This may have contributed for a large part to the non-corresponding compliance numbers of counting residual tablets after each period and recorded numbers as signed on the medication administration forms.

Lastly, planning a sufficiently long enough wash-out period requires extensive knowledge on the working dynamics of the treatment. Although we accounted for the half-life of paracetamol (on average 2.7 h), participants may have experienced a beneficial psychological effect of paracetamol that lasted throughout the second period (hence the period effect that was found). Moreover, paracetamol may not be strong enough to treat all types of pain in persons with dementia, which may explain why no differences were found between paracetamol and placebo treatment in the complete group of participants.

Compared with the distribution of males/females found in earlier research among persons with advanced dementia living in LTCF (about 72% or more female [4, 11, 19, 44]), our study had relatively fewer women (57.9%). An explanation for this could be that we included persons based on their QoL, and apparently, relatively more males had a low QoL. Also, the mean NPI total score in our research population was higher than that found in other studies in persons with advanced dementia living in LTCF (33 in our study vs. 12–16 in other studies [4, 44]). QoL may be affected to a considerable degree by neuropsychiatric symptoms, which is probably what we saw in our research population, as we selected our participants on low QoL and found relatively more neuropsychiatric symptoms.

The present study aimed to increase QOL in persons with dementia that were or were not in pain with pain treatment. No positive effects of regularly scheduled administration of paracetamol on QoL, discomfort, pain and neuropsychiatric symptoms in persons with advanced dementia were found, compared to placebo. However, there were individual cases that clearly derived benefit from paracetamol during and after the study. This could have important implications for future prescriptions of pain medication in persons with advanced dementia, and it raises questions on the statistical significance vs. the clinical relevance of the results. We showed that performing research in this vulnerable group living in LTCFs is a challenge, especially in finding the right balanced study design that accounts for this population, of which the characteristics (comorbidities and illness/death) can change quickly over a short amount of time. In addition, more research should be performed to find out which persons with dementia benefit from pain treatment, and which do not. Following this study, more attention should be paid to the compliance of medication that is administered outside a ‘unit dose package’ by a nurse/nursing assistant. Clinicians should be aware that good assessment and monitoring, and a multidomain approach instead of only prescribing pain medication, is vital for improving QoL in this vulnerable group.

## Conclusions

In this study, paracetamol did not show positive effects on QoL, discomfort, pain and neuropsychiatric symptoms in persons with advanced dementia with low QoL. It is important to find out more specifically which persons with advanced dementia could individually benefit from pain treatment with paracetamol.

## Data Availability

The datasets used and/or analysed during the current study are available from the corresponding author on reasonable request.
